# Patient perspectives on surgical handover quality: a mixed-methods survey

**DOI:** 10.1186/s13037-025-00437-z

**Published:** 2025-05-02

**Authors:** Jessica M. Ryan, Philip Tomlinson, Anastasija Simiceva, Dara O. Kavanagh, Walter Eppich, Ailbhe O’Driscoll Collins, Bevin Arthurs, Catherine Timon, Luke McGarry, Clothilde Dunleavy, Sandra Stewart, Annabella Stewart-Miller, Adam Fairhurst, Simon Roe, William Murray, Deborah A. McNamara

**Affiliations:** 1 School of Postgraduate Studies, Department of Surgical Affairs, RCSI, St. Stephen’s Green, Co. Dublin, Ireland; 2 The Bon Secours Hospital, Glasnevin Hill, Glasnevin, Dublin, Ireland; 3 Department of Surgical Affairs, RCSI, St. Stephen’s Green, Dublin, Ireland; 4 Patient & Public Partner, Dublin, Ireland; 5 Department of Surgery, Tallaght University Hospital, Tallaght, Dublin, Ireland; 6 Faculty of Medicine, Dentistry and Health Sciences, University of Melbourne, Melbourne, Australia; 7 Department of Surgery, Beaumont Hospital, Beaumont, Dublin, Ireland; 8 School of Medicine, RCSI University of Medicine and Health Sciences, Stephen’s Green, 123 St, Dublin, Ireland; 9 Office of the President, RCSI, Stephen’s Green, 123 St, Dublin, Ireland

**Keywords:** Patient and public involvement, Handoff, Surgical handover, Patient perspective, Mixed-methods

## Abstract

**Background:**

In-hospital handover of patient care is an essential but high-risk professional activity that often lacks transparency for patients. The purpose of this survey was to gain insight into surgical patients’ perceptions of handover communications between doctors, incorporating patient and public involvement to enhance accessibility and understanding.

**Methods:**

A cross-sectional, mixed-methods survey was developed with patient and public involvement and distributed to general surgery patients in two University Teaching Hospitals between 24 October 2023 and 21 July 2024. Comparative analyses of quantitative data were performed using McNemar’s test for paired nominal data and Wilcoxon rank-sum test for continuous data. Free-text responses underwent thematic analysis to validate and expand on quantitative findings. Patient and public involvement partners contributed to study design, methodology, and the final manuscript.

**Results:**

In total, 208 responses were received (52.3%). Significantly more patients reported having prior knowledge of nursing handovers (73.1%) compared to doctors' handovers (63.9*%; x*^*2*^ = 14.53, *p* = 0.0002). Patient perceptions of the handover process were generally positive; although satisfaction declined significantly with weekend handovers (*p* < 0.05). Thematic analysis identified four themes: (1) the impact of poor inter-professional communication, (2) the importance of teamwork, (3) external factors influencing handover effectiveness, and (4) patient nonchalance about their care. The use of patient and public involvement in this study improved survey accessibility and understanding of the concept and importance of handover.

**Conclusions:**

This study shows limited prior awareness of handover between doctors among surgical patients, especially the potential hazards that can arise if performed poorly. Patient and public involvement improved accessibility and understanding of the topic; however, challenges such as adequate training for meaningful engagement remain.

**Supplementary Information:**

The online version contains supplementary material available at 10.1186/s13037-025-00437-z.

## Introduction

Clinical handover is ‘the exchange between health professionals of information and responsibility for care of a patient’ and should accompany each transfer of patient care [1]. Assuming that patients are handed over at least twice per day [2, 3], it is estimated that approximately 50 million in-hospital handovers occur annually in Australia, 100 million in the United Kingdom, and 4.5 billion in China [4]. As such, handover is one of the most frequently occurring clinical communication events within hospitals.

Communication failures, which are highly preventable [5], are a leading cause of sentinel events [6], defined as incidents resulting in patient death, permanent, or severe temporary harm [7]. Clinical handover is a common source of these failures, with 40% of communication-related malpractice claims involving a failed handover [8]. The true rate of adverse events caused by inadequate surgical handover is not known; however, one-third of residents report recent patient safety events occurring as a direct result of it [9] and they score over 40% of handovers they receive as ‘less than effective’ [10]. There is currently no gold standard for surgical handover [11, 12] and practice is variable [9, 10].

Patients value transparency in interprofessional communication about their care, a lack of which can undermine trust in the healthcare team [13]. While numerous qualitative studies have evaluated the patient perspective of bedside, or ‘patient-involved’, handover [14–18], their viewpoint of provider-focused or ‘patient-uninvolved’ surgical handover has not been studied to the same degree. Even though handover often happens away from the patient [9], it is critical that their views are considered, as they remain at the centre of this important clinical communication event.

The aim of this study was to gain insights into patients’ perceptions of communication and handover between their surgical doctors. Patients and the public were involved as study partners to enhance accessibility and improve understanding of what may be considered an inaccessible and opaque professional activity.

## Methods

A cross-sectional, mixed-methods survey was distributed in paper format to patients admitted under general surgical teams in two Irish university teaching hospitals between 24 October 2023 and 21 July 2024. A patient and public partner was recruited to the core study team. The study was approved by the quality departments of both hospitals (CA2023/136 & 3714). Reporting was guided by the Consensus-Based Checklist for Reporting of Survey Studies (CROSS) [19] and the Guidance for Reporting Involvement of Patients and the Public – short form (GRIPP2-SF) [20]. The patient and public involvement member of the study team was involved in designing the survey, selecting the methodology, reviewing the pilot study findings, refining the final survey, and reviewing the results and manuscript.

### Participation of patient and public involvement (PPI) partners in survey design

As no standard survey instrument exists for this area, questions were developed through reviews of similar surveys [21, 22], interview studies [13, 23], and the Patient Measure of Safety (PMOS) [24]. The patient and public partner on the study team met with the study coordinator (JR) following the literature review and drafting of initial questions to discuss and refine the survey aims and help to develop the pilot survey draft.

This draft survey was then tested with a purposive sample of 20 in-patients (10 in each hospital), including those with reduced literacy, impaired eyesight, and those who required a parent or guardian to complete the survey. Feedback on question clarity, readability, and accessibility was sought from those who completed the survey and was recorded in contemporaneous written notes during face-to-face interviews. The results of this pilot phase were reviewed by the core study team, including the patient and public involvement partner. The same team then refined the focus and language of the final survey questions. Pilot responses were not included in the analysis. The final version of the survey contained 25 questions and can be reviewed in an additional file (Additional file 2).

### Survey administration

English-speaking patients deemed well enough for discharge by their primary inpatient team were invited to participate in the survey. Trained study team members recruited a convenience sample in-person. Those who provided verbal consent were given a paper copy of the survey, along with a QR code for online completion as per preference. Patients received clear instructions for completing both versions. Trained data collectors offered assistance in completing the survey where needed. Poster advertisements with a QR code were also put up in key areas throughout both hospitals. For the online version, it was only possible to complete the survey once from the same device. To promote recruitment, participants could provide their contact details if they wished to be entered into a competition to win a voucher.

### Quantitative analysis

Data were analysed using Stata (17.0©2021, StataCorp, Texas). Descriptive data are presented as absolute values and percentages of the total number of responses to the survey, while continuous data are presented as median (range) and mean (standard deviation, SD). Shapiro–Wilk test was used to assess normality. Comparative analyses of quantitative data were performed using McNemar’s test for paired nominal data and Wilcoxon rank-sum test for continuous data. All tests of significance were two-tailed, with a significance level of *p *< 0.05.

### Qualitative analysis

The validating quantitative data model was used. This mixed-methods triangulation approach includes open-ended questions at the end of the survey to expand on quantitative findings [25].

Qualitative analysis of responses to open-ended questions was conducted through inductive thematic analysis [26] by two investigators (JR and AS). NVivo software (NVivo qualitative data analysis software; QSR International Pty Ltd. Version 15.0.0, 2023) was used to assist with data management.

## Results

A total of 208 responses were received (*n* = 167 paper surveys, *n* = 41 web-based), representing a 52.3% response rate (total number of surveys received/total number of surveys distributed, *n* = 398). Of these, 96% (*n* = 200) were completed by patients. The remaining responses were completed on behalf of patients, including by parents and guardians (*n* = 5), carers (*n* = 1), family members (*n* = 1), or unspecified individuals (*n* = 1). The majority of participants were between 51 and 70 years old (40.4%, *n* = 84). Most patients (55.7%, *n* = 116) reported being cared for exclusively by surgical doctors during their admission, while 6.2% (*n* = 13) were unsure whether their primary team was medical or surgical. The median length of stay prior to survey completion was five days (1–98). Most patients (90.4%, *n* = 188) reported meeting 10 doctors or less during their stay; however, two reported seeing more than 20. The median rate of missing responses for each question was 3.8% (0–10.4%; Additional File 1) and data were assumed to be missing at random. Information to examine non-response error was unavailable. The final survey had a satisfactory Flesch Reading Ease Score (69.4) and Flesch-Kincaid Grade level (6.3) [27].

### Patient and Public Involvement (PPI) results

Accessibility issues identified by patients during the pilot phase included text being too small for individuals with impaired vision, difficulties faced by those with reduced literacy requiring a data collector to read the questions aloud and record responses, and the need for parents or guardians to complete the survey on behalf of younger patients. Patients also suggested improvements for survey distribution methods to enhance accessibility and participation. The patient and public involvement partner on the study team identified and helped to rectify issues with question content, order, clarity, and readability.

### Patient awareness of handover processes

Significantly more patients reported having prior knowledge of nursing handovers (73.1%) compared to doctors'handovers (63.9*%; x*^*2*^ = 14.53, *p* = 0.0002). Only 24.5% (*n* = 51) indicated that doctors explained the concept of ‘handover’ to them during their stay. Of the remaining patients, 57.1% (*n* = 84) would have liked to receive this explanation. The majority of patients (72.6%, *n* = 151) were aware that junior doctors changed shifts, although less than half (45.7%, *n *= 95) wanted to be informed when these shift changes occurred. Almost all patients (88.9%, *n* = 185) wanted to know when their care was handed over to a new consultant. Overall, patients believed that mistakes sometimes happened due to poor handovers, as reflected by a median Likert score of 3.

### Patient satisfaction with the handover process

#### Satisfaction with overall handover process

**Fig. 1:**
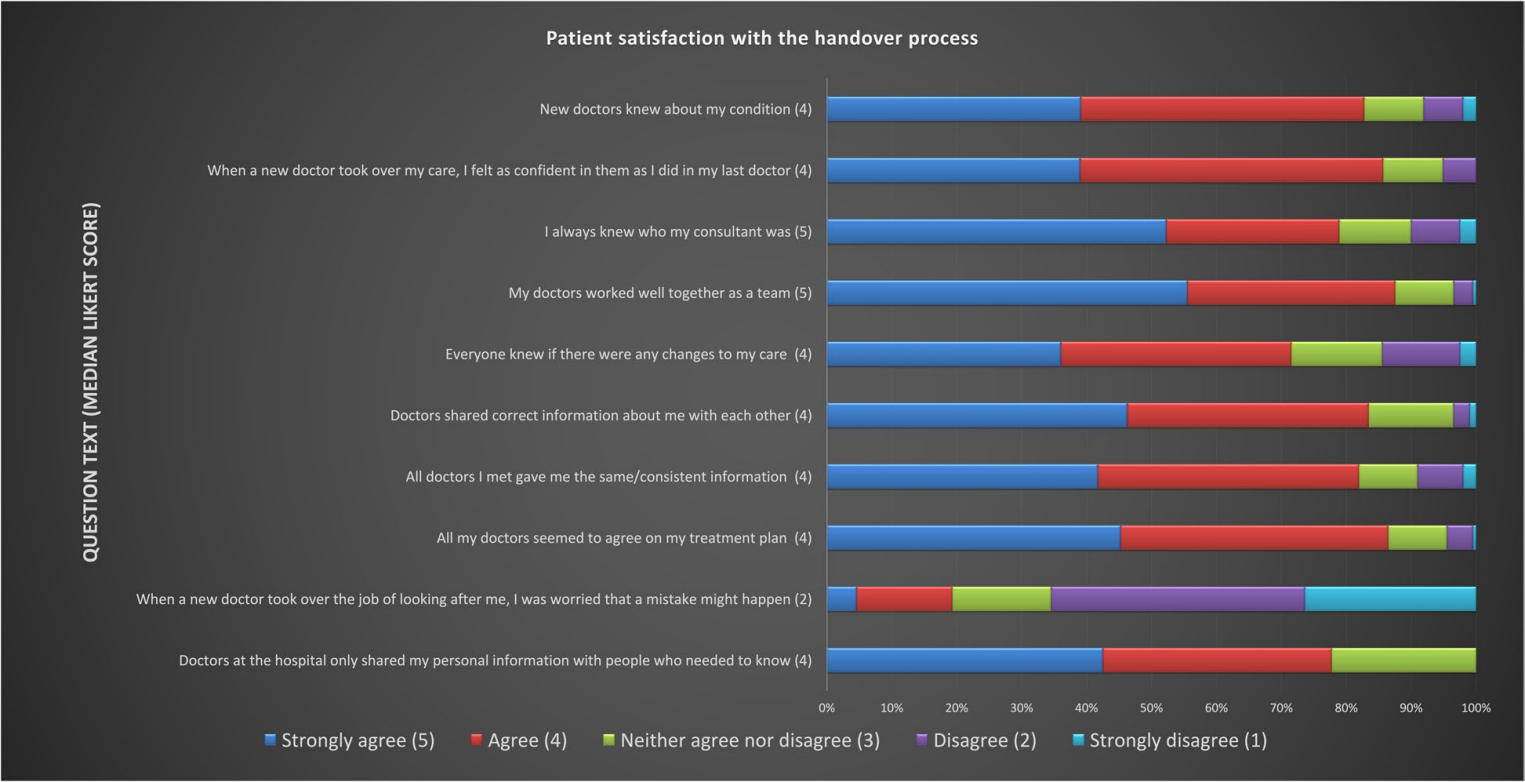
Patient satisfaction with the handover process. Distribution of patient responses (strongly agree to strongly disagree) to statements assessing satisfaction with the handover process

Patients had good perception of handover processes, agreeing or strongly agreeing with all positive statements. They felt that their doctors worked well together as a team and that they were made aware of who their consultant was (median Likert score 5: strongly agree). Overall, they were not worried about mistakes happening when a new doctor took over their care (Fig. 1; median Likert score 2—disagree).

#### Satisfaction with weekend care

Of the patients surveyed, 64.9% (*n* = 135) had a hospital stay that included at least part of a weekend. During this time, 87.4% (*n* = 118) were seen by a doctor, and 51.8% (*n* = 70) encountered a new doctor. Overall, patients reported higher satisfaction with how well new doctors understood their case the day after their admission compared with those they met on the weekend (Table 1 summarises patients’ perceptions—using a 5-point Likert scale—of new doctors’ understanding of (1) the reason for their admission, (2) their medical and surgical history, (3) their latest test results, and (4) the next steps in their treatment plan. Across all four questions, satisfaction scores were significantly higher for the day-after-admission group compared with weekend encounters (*p < 0.05, Wilcoxon rank-sum test).

**Table 1:** Patients satisfaction with new doctor knowledge the day after admission versus the weekend

	Q. The new doctors I met the day after I came to hospital knew enough about…		Q. The new doctors I met on the weekend knew enough about…		*p* value
	Mean (SD)	Median (range)	Mean (SD)	Median (range)	
The reason why I came into hospital	4.31 (0.84)	4 (1–5)	4.05 (0.92)	4 (2–5)	.0086*
My medical and surgical background	4.13 (0.99)	4 (1–5)	3.87 (0.97)	4 (2–5)	.0080*
My latest test results	4.12 (0.88)	4 (1–5)	3.88 (0.94)	4 (2–5)	.0242*
The next steps in my treatment plan	4.20 (0.82)	4 (2–5)	3.86 (0.95)	4 (2–5)	.0017*

*p 0.05, Wilcoxon rank-sum test

### Qualitative analysis

A total of 62 free-text comments pertaining to handover or communication were provided by 39 respondents. Thematic analysis identified four themes: (1) the impact of poor interprofessional communication, (2) the importance of teamwork amongst staff, (3) external factors influencing handover effectiveness, and (4) patients adopting a passive approach to their care (Table 2).

**Table 2:** Themes identified during thematic analysis

Theme	Subtheme	Example quote
The impact of poor interprofessional communication	Patients needing to repeat themselves	“I gave my history about seven times - could there be a more streamlined way?” – Respondent 95
	Patients receiving contradicting information	“i got told different things by everybody who dealt with me if i was told anything at all” – Respondent 108
	Patient distress	“I also had one doctor on the surgical team discuss a surgery with another patient beside me and while explaining the risks of the surgery reassured the patient that he wouldn't "get stuck with a colostomy bag". I was the next patient to be seen and have a permanent ileostomy. I found this very insensitive of the doctor and I'm not sure if this was because he hadn't had my details handed over yet or if he just chose his words very poorly but I found this very distressing.” – Respondent 75
	Staff miscommunication	“Often the nursing staff are passing on messages from the medical team and often the message is interpreted incorrectly. The nursing staff don’t understand/communicate rationale for decisions.” – Respondent 60
	Patients needing to correct misinformation	“As a patient I feel you have to be very knowledgeable about your condition, have basic medical knowledge and correct all medical staff when they state incorrect information which happened during my visit. I have confidence to correct people but I would be very concerned about vulnerable people who don’t speak up for themselves” – Respondent 60
	Poor staff knowledge of patients	“I’m also concerned that important and pertinent information regarding my symptoms & medical history was not written down or shared correctly with new doctors.” – Respondent 60
	Delayed or premature discharge	“I was discharged I feel prematurely. The doctor covering my care was not part of the team I believe and failed to check a drain I had attached which was not working correctly This in my view has led to complications.” – Respondent 105 “I found because I was in hospital over the bank holiday I was kept in lounger as my own consultant were not in” – Respondent 93
	Delayed treatment or medication	“I felt like I was constantly repeating myself, at one stage 3 different people were asking me about an underlying condition that delayed my surgery yet I had let them know this from the start.” – Respondent 146 “There was sometimes a delay in treatment from the nurses for dressing changes or iv meds, which would on more than one occasion meant a dose of antibiotics were missed or a dressing unchanged for hours longer than acceptable.” – Respondent 115
	Incomplete tasks	“Poor lack of communication. One doctor would say something but because it was not documented in my chart they would not do it” – Respondent 188
	Worsening of symptoms	“..but they put me on St. John's discharge unit (while waiting to transfer to a ward) in a chair for 7 hours, I couldn't sit, I told them this begore they moved me. The staff in John's put me on the resus trolley as I was in agony on the chair, I needed to lie on my side. This was incredibly distressing for such a long period of time also. The nurse in John's unit told me I should complain through PALS. She had contacted my surgical team to come and review me as I was in so much pain. No one came. When I mentioned it to my consultant the next day he shrugged and said "I never said you were able to sit". I had told them before I left [A&E]” – Respondent 146
	Prolonged ward rounds	“Every time there was a new doctor, I had to give an explanation of my history or else they took ages to look through the details in the medical folder in front of my with a big team of people when they came to check on me each morning” – Respondent 81
The importance of teamwork amongst staff	A collaborative approach to patient management	“The orthopaedic and plastic teams came up with different solutions but eventually [agreed on] the correct one for me.” – Respondent 39 “All doctors acted as one team with key goal getting me well, monitoring my progress and creating a clear recovery plan.” – Respondent 135
	Adequate staff knowledge of patients	“Everyone I spoke to on the weekend knew what was going on and I didnt need to really explain anything to anyone.” – Respondent 159
External factors impacting handover effectiveness	A lack of an electronic patient record	“When I was being admitted I was asked had I been here before- I had numerous surgeries in the hospital but my file hadn't been located” – Respondent 75 “..information created should be digital too much paper work can sometimes lead to mistakes” – Respondent 135
	A lack of staff availability around handover time	“Not enough nurses and doctors so it’s pass the buck when it comes to handover” – Respondent 23
	Accuracy of written handover	“I’m also concerned that important and pertinent information regarding my symptoms & medical history was not written down or shared correctly with new doctors.” – Respondent 60
	The impact of the weekend	“Felt like everything was a bit slower to progress on the weekends. Feels like you are waiting on your Dr and surgeon to come back to work to get any MRI/ops etc booked in.” – Respondent 137 “I found because I was in hospital over the bank holiday I was kept in longer as my own consultant were not in” – Respondent 93
Patients taking a passive approach to their care		“They should just do what they do, I have no interest in a handover being explained to me.” – Respondent 155 “The doctors knew what they were doing, I dont think they would ever make a mistake. I trust them and it does me no good to be worrying about them making mistakes. I just want to get better and leave it all to them” – Respondent 157

#### The impact of poor interprofessional communication

Patients frequently expressed frustration with having to repeat their medical history and receiving conflicting information from staff. Poor communication among staff was associated with patient distress, worsening of symptoms, and the need for patients to correct misinformation at the bedside. Concerns were also raised regarding incomplete tasks, delayed treatments, and premature discharge from hospital (Table 2).

“I was discharged I feel prematurely. The doctor covering my care was not part of the team I believe and failed to check a drain I had attached which was not working correctly.” – Respondent 105

#### The importance of teamwork

A collaborative management approach contributed to positive patient experiences. Perceptions of effective teamwork also reassured patients about staff’s familiarity with their care.

“All doctors acted as one team with key goal getting me well, monitoring my progress and creating a clear recovery plan.” – Respondent 135

“Everyone I spoke to on the weekend knew what was going on and I didn’t need to really explain anything to anyone.” – Respondent 159

#### External factors influencing handover effectiveness

Patients identified staff availability, a lack of electronic patient records, and the accuracy of written handover as factors affecting handover effectiveness. Challenges associated with hospital stays over the weekend were also reported, including unfamiliar staff, reduced frequency of patient reviews, limited staff availability, and delays in investigations and procedures.

“..information created should be digital too much paper work can sometimes lead to mistakes” – Respondent 135

“Felt like everything was a bit slower to progress on the weekends. Feels like you are waiting on your [doctor] and surgeon to come back to work to get any MRI/ops etc booked in.” – Respondent 137

#### Nonchalance about their care

Some patients expressed disinterest in understanding or engaging with the handover process, preferring to entrust this professional activity entirely to the medical team. One patient stated:

“They should just do what they do, I have no interest in a handover being explained to me.” – Respondent 155

“The doctors knew what they were doing, I don’t think they would ever make a mistake. I trust them and it does me no good to be worrying about them making mistakes. I just want to get better and leave it all to them” – Respondent 157

## Discussion

This cross-sectional, mixed-methods survey of surgical patients in two University Teaching Hospitals revealed a low level of patient awareness regarding doctors’ handover, despite positive perceptions of the handover process. However, satisfaction with handover processes was negatively influenced when the hospital stay included a weekend. Qualitative analysis of free-text comments provided deeper insights into the adverse impacts of poor interprofessional communication on patients and emphasised the benefits of a collaborative approach to management. The use of patient and public involvement in this study helped to ensure that the survey was accessible, and that the concept of handover, a professional activity which can lack transparency, was understandable to the public.

This study is unique in its goal to evaluate patient perceptions of clinical handover in collaboration with patient and public partners, with only one previous study reported [28]. According to INVOLVE, patient and public involvement involves research conducted with or by members of the public rather than to, about, or for them [29]. Given the significant impact of poor handover on patients [30] and the potential challenges in understanding the concept, the authors assert that patient and public involvement is crucial for studies in this area. However, it can present challenges, particularly when participants feel unable to contribute effectively due to a lack of training or preparation [31]. This may be especially relevant for complex topics like handover, where some patients may lack interest or fail to fully appreciate the associated risks, as suggested by the qualitative findings above. Despite these challenges, other researchers have demonstrated success in enabling patients and the public to meaningfully contribute to consensus-building processes in specialised areas [32]. Future patient and public involvement efforts in clinical handover should prioritise meaningful involvement by providing patients with adequate support to ensure they fully understand the process of handover and its implications [33].

Patients demonstrated a good understanding of the impacts of poorly performed handovers when they directly affected them. These findings align with previous literature, highlighting issues such as poor staff knowledge of patients [34], delayed treatment [35], and needing to repeat information with new staff members [14]. Interestingly, patients also demonstrated an understanding of external factors impacting handover, such as the need for adequate staffing, a finding similarly reflected in prior research [28]. Weekend care had a notable impact on patient experience, as demonstrated by both quantitative and qualitative findings, highlighting the importance of standardized handovers across all transitions of care [36].

Patient respondents were only from two hospitals, which may limit generalisability of the findings; however, previous work has drawn from a similar population [21, 22]. Additionally, the sample of patients who provided free text comments was even smaller. Future work should include an in-depth semi-structured interview approach to gain rich insights into patient perspectives. Additionally, while patient and public involvement has clear benefits during all study stages [37], it was not employed in the analysis of these results, as training in statistical methods and thematic analysis would need to be provided, and the study team did not have resources available to provide this training. This is a known challenge faced by researchers using patient and public involvement [31].

## Conclusion

Patients have limited prior awareness of handover between doctors, especially the potential hazards that can arise if performed poorly. Weekend care negatively impacted satisfaction, underscoring the need for improved and standardised handover processes during out-of-hours periods. Patient and public involvement improved accessibility and understanding of the topic; however, challenges such as adequate training for meaningful engagement remain.

## Supplementary Information

Additional file 1. Copy of survey. This is a PDF copy of the survey used for this study, which details survey instructions, questions, answer options, and further information.

Additional file 2. Quantitative survey responses. All quantitative survey responses, response rates, and missing data for each question are included in this file.

## Data Availability

The quantitative dataset supporting the conclusions of this article are included within the article and its additional files.

## Abbreviations

CROSS: Consensus-Based Checklist for Reporting of Survey Studies
GRIPP2-SF: Guidance for Reporting Involvement of Patients and the Public – short form
MPS: Medical Protection Society
PMOS: Patient Measure of Safety
PPI: Patient and public involvement
RCSI: Royal College of Surgeons in Ireland
SD: Standard deviation
