# Supplementary material for: Patient perspectives on surgical handover quality: a mixed-methods survey
Source: Patient Saf Surg. 2025 May 2;19:14. doi: 10.1186/s13037-025-00437-z (PMC12049061; doi:10.1186/s13037-025-00437-z)
Supplement: Supplementary file 2 — Additional file 2. Quantitative survey responses. All quantitative survey responses, response rates, and missing data for each question are included in this file. [file 13037_2025_437_MOESM2_ESM.pdf]

Survey Information Sheet

Why is this survey being done?

- **'Handover' is when one doctor passes a patient's care to another doctor.**
- **This can happen when a doctor is finishing their shift.**
- **At handover, the outgoing doctor tells the incoming doctor about the patient's condition, like their symptoms, tests, and treatments.**
- **The incoming doctor might not know about their new patients until they get this information.**
- **A good handover is important because it makes sure no important details are missed and helps patients get better care. It keeps patients safe.**

Why am I being asked to do this survey?

- **We're asking you to take this survey because you were in hospital recently and doctors who work in surgery took care of you.**
- **When answering, please think about that recent hospital visit.**
- **Your answers will help us understand how you feel about how doctors talk to each other about your care.**
- **Your feedback could help to make those talks between doctors better.**

How do I complete this survey?

- **This survey should be completed by the person who was admitted to hospital, however, it can also be completed for them by their carers/guardians/family members if needed.**
- **Try to fill out this survey on the day you leave the hospital or as close to this day as possible.**
- **Take time to read each question. The survey will take you about 10 minutes.**
- **Please be as honest as you like - this survey is completely anonymous.**

Your details

The following questions will ask you for some of your details.
There is no way to identify you from the information you give.

1. **Which of the following describes you:**

- ◯ The patient
- ◯ Parent or guardian of the patient
- ◯ Family member of the patient
- ◯ Carer for the patient
- ◯ Translator
- ◯ Other

Other (please specify)

[ ]

All of the following questions relate to the patient who was admitted to hospital:

2. **Which hospital were you recently admitted to?**

- ◯ Tallaght Hospital
- ◯ Beaumont Hospital

3. **How old are you?**

- ◯ Under 20
- ◯ 20-30
- ◯ 31-40
- ◯ 41-50
- ◯ 51-60
- ◯ 61-70
- ◯ 71-80
- ◯ 81-90
- ◯ 91 or older

4. **Your gender:**

- ◯ Male
- ◯ Female
- ◯ Prefer not to say
- ◯ Other (please specify)

[ ]

5. **Who were the main doctors taking care of you during your recent hospital visit?**

- ◯ Surgical doctors (doctors who carry out surgery)
- ◯ Medical doctors
- ◯ Both
- ◯ I don’t know.
- ◯ Other (please specify)

6. **What were the total days for this hospital visit so far?**

7. **How many different doctors did you meet during your hospital visit?**

- ◯ 5 or less
- ◯ 6-10
- ◯ 11-15
- ◯ 16-20
- ◯ More than 20

Your perspectives - page 1 of 2

8. **Were you aware of <u>doctors</u>' handovers before this survey?**

- ◯ Yes
- ◯ No

9. **Were you aware of <u>nurses</u>' handovers before this survey?**

- ◯ Yes
- ◯ No

10. **Did you know that junior doctors (i.e., not consultants) rotated in shifts to care for you during your time in hospital?**

- ◯ Yes
- ◯ No

11. **During your hospital visit, did any doctors explain to you what a "handover" was?**

- ◯ Yes
- ◯ No

12. **If they didn't explain, would you have liked them to tell you about "handover"?**

- ◯ Yes
- ◯ No
- ◯ Does not apply, they did explain it to me.

13. **Let us know how much you agree with these statements about your hospital visit.**

If it is difficult to choose one answer, think about your overall visit.

	Strongly agree	Agree	Neither agree nor disagree	Disagree	Strongly disagree
New doctors knew about my condition	○	○	○	○	○
When a new doctor took over the job of caring for me, I felt as confident in them as I did in my last doctor	○	○	○	○	○
I always knew who my consultant was	○	○	○	○	○
My doctors worked well together as a team	○	○	○	○	○
Everyone knew if there were any changes to my care	○	○	○	○	○

If you want to share any comments about these questions, please put them here:

14. **Let us know how much you agree with these statements about your hospital visit.**

If it is difficult to choose one answer, think about your overall visit.

	Strongly agree	Agree	Neither agree nor disagree	Disagree	Strongly disagree
Doctors shared correct information about me with each other	○	○	○	○	○
All doctors I met gave me the same/consistent information	○	○	○	○	○
All my doctors seemed to agree on my treatment plan	○	○	○	○	○
When a new doctor took over the job of looking after me, I was worried that a mistake might happen	○	○	○	○	○
Doctors at the hospital only shared my personal information with people who needed to know	○	○	○	○	○

If you want to share any comments about these questions, please put them here:

15. **Please think about when you came into the hospital and indicate your agreement with the following:**

"The new doctors I met the *day after I came to hospital* *knew enough about…"*

	Strongly agree	Agree	Neither agree nor disagree	Disagree	Strongly disagree
The reason why I came into hospital	○	○	○	○	○
My medical and surgical background	○	○	○	○	○
My latest test results	○	○	○	○	○
The next steps in my treatment plan	○	○	○	○	○

If you want to share any comments about these questions, please put them here:

16. **During your stay, were you in hospital for all or part of a weekend?**

- ○ Yes
- ○ No

Your perspectives - page 2 of 2

17. **Were you seen by a doctor over the weekend?**

- Yes
- No
- I don’t know.
- Does not apply

18. **Did you meet any new doctors over the weekend?**

- Yes
- No
- I don't know
- Does not apply

19. **If you spent all or part of a weekend in the hospital. Please indicate your agreement with the following:**

“The new doctors I met on the weekend knew enough about...”
(You may skip this question if you were not seen by a doctor on the weekend)

	Strongly agree	Agree	Neither agree nor disagree	Disagree	Strongly disagree
The reason why I came into hospital	◯	◯	◯	◯	◯
My medical and surgical background	◯	◯	◯	◯	◯
My latest test results	◯	◯	◯	◯	◯
The next steps in my treatment plan	◯	◯	◯	◯	◯

If you want to share any comments about these questions, please put them here:

20. **How often do you think mistakes happen because of poor handover? (i.e., information is not given correctly from one doctor to another)**

- Never
- Rarely
- Sometimes
- Often
- Always
- I don’t know

21. **Should you be told when your junior doctor changes shift?**

- ◯ Yes
- ◯ No

22. **Should you be told when a new consultant takes over the job of caring for you?**

- ◯ Yes
- ◯ No

Final details and comments

* 23. **Which date did you complete this survey?**

Date

Date

DD/MM/YYYY

24. **Any additional comments:**

25. **If you, or someone you care about, have had a bad experience in hospital that you felt was caused by poor communication between your doctors, you can share your story below, if you wish.**

We will always make sure that it is not possible to identify you from your story.

Study contact details and further information

Future contact

If you would like to take part in future studies to help improve communication between doctors, or to discuss your story in further detail, please email surgicalhandover@rcsi.com.

We cannot manage or reply to messages with complaints or concerns about medical treatment. Any messages we receive like this will be deleted without a response so that we can protect your personal information.

If you have a complaint or concern you wish to make with the hospital, you can contact the following Patient Advisory Liaison services instead.

Note: These services can also be used to give positive feedback:

Tallaght Hospital

Website: https://www.tuh.ie/Patient-Advice-Liaison-Service/
Phone number: 01 414 4709
Email address: PALS@tuh.ie
Service Hours: Monday to Friday 9am-5pm

Beaumont Hospital

Website: http://www.beaumont.ie/pals/
Phone number: (01) 809 3234 / 2427 (24-hr answer machine)
Email address: PALS@beaumont.ie
Service Hours: The office is open from Monday to Friday 10:00 to 12:00 and 14:00 to 16:00

Competition to win one of two €100 one-for-all vouchers

Please follow these steps if you would like to enter this competition:

1. Copy the link below
2. Complete the survey by clicking 'Done' (see button below)
3. Paste the link into your browser - this will take you to a separate survey to enter your details

https://www.surveymonkey.com/r/BMBDKWD

If for any reason you lose the link, you can email the study team at surgicalhandover@rcsi.com with your details and we will enter them for you. You have also been provided with a paper copy to enter your details, which can be dropped into the box on your ward.
